# Towards an Integrative, Eco-Evolutionary Understanding of Ecological Novelty: Studying and Communicating Interlinked Effects of Global Change

**DOI:** 10.1093/biosci/biz095

**Published:** 2019-09-18

**Authors:** Tina Heger, Maud Bernard-Verdier, Arthur Gessler, Alex D Greenwood, Hans-Peter Grossart, Monika Hilker, Silvia Keinath, Ingo Kowarik, Christoph Kueffer, Elisabeth Marquard, Johannes Müller, Stephanie Niemeier, Gabriela Onandia, Jana S Petermann, Matthias C Rillig, Mark-Oliver Rödel, Wolf-Christian Saul, Conrad Schittko, Klement Tockner, Jasmin Joshi, Jonathan M Jeschke

**Affiliations:** 1 University of Potsdam, Biodiversity Research/Systematic Botany, Potsdam, Germany; 2 Technical University of Munich, Restoration Ecology, Freising, Germany; 3 Berlin-Brandenburg Institute of Advanced Biodiversity Research (BBIB), Berlin, Germany; 4 Freie Universität Berlin, Institute of Biology, Berlin, Germany; 5 Swiss Federal Research Institute WSL, Forest Dynamics, Birmensdorf, Switzerland, also with the Leibniz Centre for Agricultural Landscape Research (ZALF), Müncheberg, Germany; 6 Leibniz Institute for Zoo and Wildlife Research, Berlin, Germany and the Freie Universität Berlin, Department of Veterinary Medicine, Berlin, Germany; 7 University of Potsdam, Institute of Biochemistry and Biology, Potsdam, Germany; 8 Leibniz-Institute of Freshwater Ecology and Inland Fisheries (IGB), Berlin, Germany; 9 Museum für Naturkunde – Leibniz Institute for Evolution and Biodiversity Science, Berlin, Germany; 10 Technische Universität Berlin, Department of Ecology, Ecosystem Science/Plant Ecology, Berlin, Germany; 11 ETH Zurich, Institute of Integrative Biology, Zurich, Switzerland; 12 Stellenbosch University, Centre for Invasion Biology (CIB), Department of Botany and Zoology & Department of Mathematical Sciences, Matieland, South Africa; 13 UFZ – Helmholtz Centre for Environmental Research GmbH, Department of Conservation Biology, Leipzig, Germany; 14 University of Salzburg, Department of Biosciences, Salzburg, Austria; 15 Austrian Science Funds – FWF, Vienna, Austria; 16 Institute for Landscape and Open Space, HSR Hochschule für Technik, Rapperswil, Switzerland

**Keywords:** Anthropocene, eco-evolutionary experience, global change, novel ecosystems, shifting baselines

## Abstract

Global change has complex eco-evolutionary consequences for organisms and ecosystems, but related concepts (e.g., novel ecosystems) do not cover their full range. Here we propose an umbrella concept of “ecological novelty” comprising (1) a site-specific and (2) an organism-centered, eco-evolutionary perspective. Under this umbrella, complementary options for studying and communicating effects of global change on organisms, ecosystems, and landscapes can be included in a toolbox. This allows researchers to address ecological novelty from different perspectives, e.g., by defining it based on (a) categorical or continuous measures, (b) reference conditions related to sites or organisms, and (c) types of human activities. We suggest striving for a descriptive, non-normative usage of the term “ecological novelty” in science. Normative evaluations and decisions about conservation policies or management are important, but require additional societal processes and engagement with multiple stakeholders.

Science has made great advances in identifying the currently ongoing major environmental changes and the underlying human activities (e.g., Ellis 2011, Barnosky et al. 2012, Sullivan et al. 2017, Vellend et al. 2017). Several disciplines, such as climate-change ecology, invasion ecology, restoration ecology, disease ecology, and urban ecology have emerged and address how environmental changes affect organisms and ecosystems, and how they can be mitigated. However, many important human-driven changes have remained remarkably understudied. Examples include the mass release of synthetic chemicals into the environment (Bernhardt et al. 2017), landscape-scale topographical changes, for example through the creation of artificial islands (Li et al. 2014), mountain-top removal for coal mining (Lutz et al. 2013), and urban expansion, or the cascading effects of soil and aquatic microbes in changing environments (Ricciardi et al. 2017) (box 1). All these changes—be they poorly investigated or better explored—are interlinked, occur simultaneously and at accelerating rates (Steffen et al. 2015, Waters et al. 2016). Whereas the ecological effects of some single global-change elements are comparatively well studied, our understanding of their combined effects and their complexity remains limited (Kueffer 2015, Pendleton et al. 2016). This poses a major challenge for scientists. Indeed, explaining and predicting the synergistic, additive, and antagonistic effects of multiple drivers on organisms and ecosystems requires coordinated cross-disciplinary research approaches, supported by a shared understanding of key terms. An integrative conceptual framework is thus needed to address interlinked effects of global change.

**Box 1. Novelty in microbial communities: Highly relevant but poorly studied**

Global change strongly affects microbes and microbial communities. Microbes are increasingly transported around the world, and instances where novel pathogens have entered a community have occurred throughout human history. Also, intentional transportation of non-pathogenic microbes, for example of mycorrhizal fungi as inoculum for agricultural application, is happening increasingly, with largely unknown consequences (Schwartz et al. 2006). Regional or global transportation processes often involve the unintentional transportation of entire microbial communities, for example, in ballast water or on living goods. As a consequence, incidences of coalescence of previously separated microbial communities (Rillig et al. 2015) are widespread.

Changing selective pressures on existing microbes can lead to the evolution of novel organisms. The massive increase in antibiotic use, particularly in agricultural settings, has driven the rise of novel microbes resistant to most natural and synthetic antimicrobial agents. Such processes have increased since the 17th century (Duggan et al. 2016).

Changing the composition of microbial communities can have cascading effects in ecosystems. Microbes strongly affect the fitness of organisms as well as trophic interactions between plants and herbivores (Hird 2017) and even between herbivores and carnivores (Dicke and Hilker 2003). For example, the microbial players associated with roots and leaves impact the nutritional quality of plants (Friesen et al. 2011). Hence, quantitative or qualitative alterations of the microbiome associated with organisms can have enormous effects on entire food webs and can even drive speciation (e.g., Hird 2017).

These examples highlight the potential significance of novelty in microbes and microbial communities. With the exception of emerging pathogens, though, novelty is not an explicit focus of current microbial ecology (Yakob 2013). One likely reason is the challenge to apply the concept to microbial systems. Novel populations of microbes arise continually during ecological timescales as a consequence of relatively rapid microbial lifecycles and the presence of core and ancillary genomes. Also, horizontal gene transfer can be quite common within microbial communities and thus lead to widespread novel genotype combinations. Due to incomplete molecular surveys coupled with very high levels of microbial diversity and variability, reference states of microbial diversity are generally unknown. And it is quite clear that microbes respond to habitat alterations on a drastically different scale than macro-organisms (Veresoglou et al. 2015). We believe, however, that these challenges should be overcome, because global-change effects on ecosystems can only be fully understood if microbes and microbial communities are considered.

Many tools offered above for studying ecological novelty can be applied in microbial ecology. For example, applying the site-specific perspective, the belowground microbial community of an ecosystem that has been classified as novel based on vegetation could be compared to that of a reference system. This would allow assessment of whether different components of an ecosystem correspond to each other in terms of their novelty, or whether responses to global change are uncoupled (e.g., Adair et al. 2019). By applying the organism-centered perspective, the effects of a novel biotic or abiotic environment on microbes and their communities can be studied (see e.g., Ramirez et al. 2019). With our contribution, we hope to stimulate research on ecological novelty in sub-disciplines of biology that so far have not systematically considered global-change effects. Microbial biology could be one of these sub-disciplines. Performing research under the umbrella of ecological novelty would enhance knowledge transfer from other disciplines (e.g., invasion science, urban ecology) to microbial ecology and vice versa. As a result, we expect a strong increase in our abilities to understand, manage, and mitigate global-change effects.

To describe profound and often unprecedented transformations of ecosystems as a consequence of species invasions, major transformations by human land use, or climate change, Hobbs and colleagues introduced the concept of “novel ecosystems” (see Glossary and box 2) a decade ago (Hobbs et al. 2006, Hobbs et al. 2009, Hobbs et al. 2013b, building on prior ideas e.g., by Milton 2003). Since then, this term and related ones such as “emerging ecosystems”, “novel communities”, and “novel organisms” have been increasingly used to describe and investigate far-reaching ecological shifts in response to human-induced environmental change (box 2). The concept has been embraced by many ecologists, but it also sparked discussions on the normative meaning and the management goals for anthropogenically modified ecosystems. Critics fear that the term and its underlying ideas may open the doors to impunity and put previous political achievements of nature conservation at risk, whereas proponents emphasize its usefulness for broadening the possibilities of conservation efforts (Marris et al. 2013, Murcia et al. 2014, Kattan et al. 2016, Miller and Bestelmeyer 2016).

**Box 2. Roots of the term ecological novelty**

**Novel ecosystems**

Hobbs and colleagues suggested that novel ecosystems are of increasing importance, especially within the field of restoration ecology (Hobbs et al. 2009). They differentiated between historical, hybrid, and novel ecosystems (Hobbs et al. 2009, Hobbs et al. 2013a). “Hybrid systems” are no longer in their historic state but can develop towards historic conditions after the cessation of human impacts or by restoration efforts. If certain thresholds are passed, this return is no longer possible, and the system irreversibly transforms to a novel ecosystem. Since its introduction, the term “novel ecosystems” has been used to describe a diversity of man-made or modified systems such as urban ecosystems (Kowarik 2011), agricultural areas (Hobbs et al. 2006), afforested fields (Juutilainen et al. 2016), invaded or urban wetlands (Thomasen and Chow-Fraser 2012), regulated streams (Moyle 2014), restored post-mining sites (Laarmann et al. 2015), tree plantations (Lindenmayer et al. 2015), private yards (Knapp et al. 2012), green roofs (Holt 2016), and gaps between buildings (Kajihara et al. 2016). Some authors have used other terms to describe similar systems, for example “emerging ecosystems” (Milton 2003), “anthromes” (Ellis 2013) or “domesticated ecosystems” (Tockner et al. 2011).

**Previous definitions of ecological novelty**

In evolutionary biology, the term ecological novelty has been used to describe unprecedented situations such as newly evolved species traits that have enabled new ecological functions, or abiotic change that has led to new (“novel”) situations triggering evolution (e.g., Zhang et al. 2010). Saul and colleagues promoted ecological novelty as tightly linked to eco-evolutionary experience (Heger et al. 2013, Saul et al. 2013, Saul and Jeschke 2015): an organism is facing novelty if its environment (including its interaction partners) differs from the environment it evolved in, thus rendering the “experience” it has accumulated during its evolution only partly applicable (see also Sih et al. 2011, McDonnell and Hahs 2015). In contrast, Radeloff and colleagues (Radeloff et al. 2015) defined novelty as “the degree of dissimilarity of a system, measured in one or more dimensions relative to a reference baseline, usually defined as either the present or a time window in the past”. A broader definition was proposed by Kueffer (2015) who suggested that ecological novelty affects all levels of biological organisation, from genomes to landscapes. Our framework builds on these and several other previously raised ideas, integrating them into an umbrella concept (figures 1, 3).

**Figure 1.**
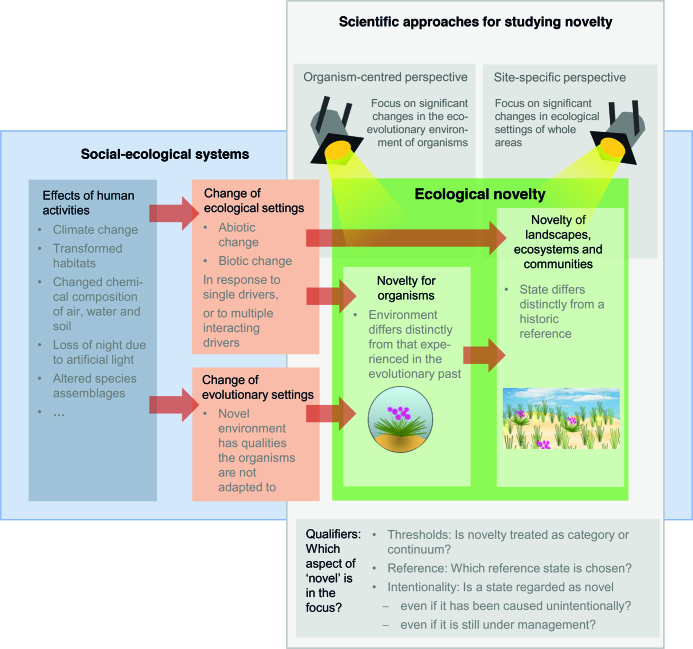
Ecological novelty as an umbrella concept for studying ecological and evolutionary effects of global change (green box). Red arrows depict simplified causal chains within social-ecological systems (light blue box), leading from human activities to the creation of ecological novelty. The light grey box highlights the focus of this paper in this causal chain. Dark grey boxes indicate that diverse approaches are needed to capture all relevant aspects of ecological novelty. The choice of a study system and research method (see figure 2 step 1) requires the specification of a research perspective (top) and qualifiers (bottom dark grey box).

The concept of novel ecosystems mainly addresses the ecosystem and landscape level. It has also been applied at the community level and has been used to analyze effects of novel ecosystems on single organisms (see e.g., Harris et al. 2013). Yet, the concept neither captures the population paradigm *sensu* Pickett et al. (2007) in its full breadth, nor does it provide an evolutionary perspective on organisms. This is a critical conceptual shortfall since urgent questions beyond the scope of the established novel ecosystems concept remain, for example: Which elements of global change other than climate change, biological invasions and urbanization have significant short- and long-term effects on organisms and ecosystems? How does the interaction of diverse elements of global change affect ecology and evolution? How do global-change effects at the organism level influence higher organizational levels such as populations, communities, and ecosystems? Which cascading effects on microbes have repercussions at the ecosystem level? Another limitation of working with the concept of novel ecosystems is that the term has often been loaded with a normative meaning (“embracing novelty” as the “new normal”; Marris 2010, Vince 2011). By contrast, the concepts of novel communities and novel organisms (see Glossary) have been used as rather value-neutral terms.

Here, we propose to use ecological novelty as an integrative, descriptive umbrella term. Building on the concept of novel ecosystems, related ideas, and previous definitions of ecological novelty (box 2), we present a conceptual framework to better describe, understand, predict, and communicate the wide range of consequences that environmental change has for organisms, ecosystems and landscapes (figure 1). Human activities can have profound effects on ecological and evolutionary settings (e.g., Collins et al. 2011, Díaz et al. 2015, Ellis 2015, Sullivan et al. 2017), which in turn can lead to the creation of novel landscapes, ecosystems, and communities, and novel situations for organisms (red arrows in figure 1). Ecological novelty encompasses all these effects (green box in figure 1). We here argue for “ecological novelty” as a broad concept that (a) covers studies from organisms to ecosystems, and (b) consolidates diverse methodological approaches of studying ecological as well as evolutionary effects of human activities within ­social-ecological systems.

Our focus in the following is on how ecological novelty affects organisms, populations, communities, ecosystems, and landscapes, and how organisms trigger novelty (grey box in figure 1). Research on ecological novelty is additionally, and often not transparently, linked to societal values and goals (Backstrom et al. 2018) (figure 2). These links to the societal dimension are especially apparent during the phases of initiating a study (step 1 in figure 2) and of evaluating and applying its results (i.e., when deriving management decisions and implementing management measures, steps 3 and 4 in figure 2). Thus, scientific findings may be influenced by implicit normative assumptions. However, for deriving arguments from these findings about how something ought to be, it is still necessary to separate the empirical (“factual”) results from their normative evaluation (e.g., to avoid a naturalistic fallacy). In this sense, we here suggest using the term ecological novelty in a descriptive rather than normative way. This will allow for a case-specific decision on whether novelty is or is not in line with societal goals (see Backstrom et al. 2018).

**Figure 2.**
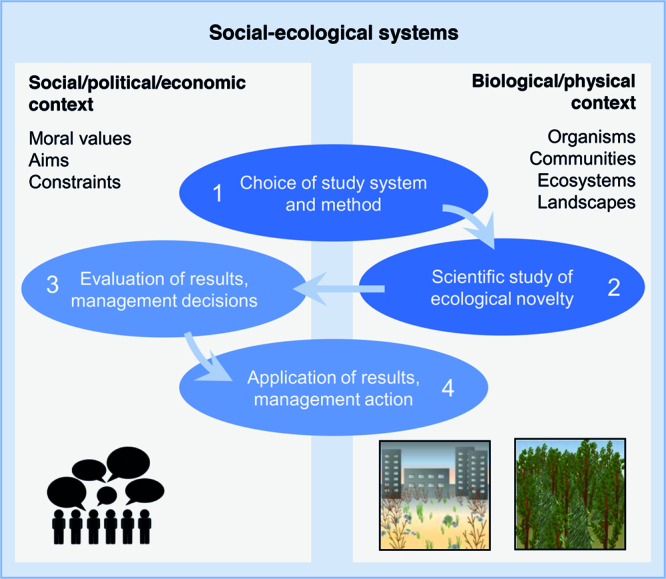
Simplified illustration of research steps with a focus on ecological novelty. The focus of this paper are steps 1 and 2 (dark blue). Whereas steps 1 and 4 are strongly influenced by both the social-economic and the bio-physical context, step 2 is ideally descriptive, avoiding biases stemming from the social, political, or economic context. We suggest using the term “ecological novelty” mainly in this context, that is, without a normative connotation. The pictures at the bottom right illustrate two possible objects of a scientific study along a gradient of novelty (cf. figure 3).

## Site-specific versus organism-centered perspectives on ecological novelty

Ecological novelty, as we define it, encompasses the concepts of novel organisms, novel communities, novel ecosystems, as well as novel selection pressures such as novel interactions and novel abiotic conditions. The colloquial term “novel” describes something that is different from everything that was there before (cf. Princeton University 2010); it does not *per se* include an evaluation as to whether this difference is negative or positive. Based on this colloquial meaning, the concept of ecological novelty has to include two components: (1) a change-dependent (“different”) and (2) a time-dependent (“before”) component. Both components require the consideration of reference conditions (see below).

When applying this broad framework to global-change effects on organisms and ecosystems, various options remain for a given study as to how exactly ecological novelty can be addressed. Choosing from these options is part of the first step in research on ecological novelty, that is, the choice of a study system and method (figure 2, step 1). We identified two major, complementary approaches for investigating ecological novelty paralleling the two paradigms in ecology (ecosystem and population paradigm, Pickett et al. 2007 p. 11) as well as the dual focus of nature conservation on places and species (Hobbs et al. 2018): a site-specific versus an organism-centered approach. Framing these two perspectives conceptually is an important step towards clarifying and focusing scientific and public debates in this domain, and for understanding the multiple facets and interdependencies of global-change effects. Previously, these two perspectives have not been integrated into a single framework; doing this allows the addressing of ecological and evolutionary consequences of global change in a comprehensive and integrative way.

Rather than prescribing a single path for future research on ecological novelty, we encourage multiple, complementary lines of research. We thus provide a toolbox for researchers on ecological novelty who can, depending on their research question and system, choose (1) the site-specific or organism-centered perspective described in the following paragraphs as well as (2) three qualifiers (thresholds, reference conditions, and intentionality) described in the next section.

The site-specific perspective focuses on human-induced changes that lead to abiotic or biotic alterations at a specific site. The concept of novel ecosystems as introduced by Hobbs and colleagues (Hobbs et al. 2006, see box 2) has been formulated from this perspective, and recent research on “climatic novelty” (Ordonez et al. 2016) uses this approach as well. The required reference conditions are usually defined as historic conditions at a site (see table 1 for additional characteristics of this approach). Research performed from this perspective asks, for example, how global change induces novelty at a site, in an ecosystem, or in a community, and how such novel systems can be managed.

**Table 1.** Characterization of the two complementary perspectives that can be taken when studying and managing ecological novelty.

	Site-specific perspective	Organism-centered perspective
Definition of novel	The state of a focal area or site is novel if it distinctly differs from a reference specified based on historic criteria, i.e., if the current conditions differ from a suggested historic state (cf. Radeloff et al. 2015). A site can be novel with respect to biotic features (e.g., species assemblages) or other environmental factors (e.g., soil), or both.	A focal species is experiencing a novel environment if the latter distinctly differs from the environment in the focal species’ evolutionary past, i.e., if the focal species lacks eco-evolutionary experience. Novelty can occur in species interactions or with respect to abiotic environmental conditions.
Opposite of novel	“Ancient” or “historic” states (e.g., natural remnants, restored areas).	Situations within the range of the species’ eco-evolutionary experience; they can be called “known”, “analog” or “familiar”.
Reference conditions	Reference conditions are chosen based on historic criteria, e.g., on knowledge about the past development of the site.	Reference conditions are chosen based on eco-evolutionary criteria, e.g., on knowledge about ecological conditions during the evolutionary past of the focal species.
	Example 1: Conditions in the focal area before the last glaciation; e.g., in beech-dominated forest ecosystems in Central Europe.	Example 1: Environment of a resident species (a “stayer” according to Hobbs et al. 2018) prior to some major environmental change (e.g., draining of a wetland).
	Example 2: Conditions at a comparable “near-natural” site (e.g., a forest remnant) as a proxy for a state prior to man-made change.	Example 2: The biotic and abiotic conditions in the native range of an alien species.
Time dependence	The classification of a focal area as novel or its positioning along a novelty gradient can change if the historic reference is exchanged (shifting baseline phenomenon, see main text).	From the viewpoint of a focal species, novelty is transient and can erode: the longer an organism experiences the novel condition, the more it will have an opportunity to adapt, and the less novel the condition will be—it will become “familiar”.
Relation to societal values and management goals	A management goal can be the conservation or restoration of ancient or historic conditions at a site (e.g., grazed heathland), or the conservation or initiation of conditions that were not intentionally designed (e.g., urban wildness, see figure 3a). In the latter case, novel conditions can be compatible with management goals.	If biodiversity conservation and management aim at creating optimal conditions for a focal species (e.g., a rare species), the goal can be to provide known or analog conditions for this species (e.g., by introducing analog seed dispersers replacing extinct species), irrespective of these conditions being intentionally designed or natural (see figure 3b).
Main area of application	Description, explanation, and prediction of changes in ecosystems and landscapes in response to global change; support for priority setting and action on environmental policies and management of an area (e.g., conservation, restoration or creation).	Description, explanation, and prediction of the impact of global change on organisms; support for identifying biodiversity-related goals, e.g., aimed at the management of endangered species or potentially endangered “stayers” (Hobbs et al. 2018) facing global change (figure 3b).

Complementary to the site-specific perspective, the organism-centered perspective considers whether and how organisms are affected by abiotic or biotic human-induced change, for example, in their morphological traits, behavior, or fitness (Sih et al. 2011, McCarthy et al. 2017). The basic idea is that abiotic and biotic environmental conditions will be perceived as novel by a focal organism if they are outside the range of environmental conditions experienced during a species’ evolutionary history (see e.g., Wilsey et al. 2011, Saul et al. 2013). Abiotic factors and past interactions with competitors, predators, prey, or parasites determine a species’ eco-evolutionary experience (Heger et al. 2013, Saul et al. 2013) or “adaptedness” (McDonnell and Hahs 2015). A mismatch between this experience and the conditions an organism is exposed to represent novelty (Heger et al. 2013, Saul et al. 2013, see also Sih et al. 2011; box 2). This might occur if an organism interacts with domesticated or cultivated organisms, or if it is translocated beyond the species’ previous biogeographical range. An environmental condition can thus be novel for organisms of one species, but familiar (i.e., within the range of their eco-evolutionary experience) for organisms of another species (table 1). This research perspective does not focus on how an organism drives the novelty of a site; instead, it asks how novel an environment is for an organism, and how this novelty affects the organism.

## Three qualifiers: thresholds, reference conditions, and intentionality

Stating whether the site-specific or the organism-centered perspective has been chosen helps in clarifying what exactly is meant by “ecological novelty”. When choosing the system and research method for studying ecological novelty (figure 2), it is helpful to make use of three qualifiers: (1) thresholds, (2) reference conditions, and (3) intentionality.

### Thresholds: novelty as category or continuum?

The novelty of a site or of a species’ environment can be assessed using either pre-set criteria describing a continuous gradient (e.g., dissimilarity of community-composition patterns or interaction networks) or pre-defined thresholds (e.g., Goring et al. 2016, Leon et al. 2016). In the concept of novel ecosystems as presented by Hobbs et al. (2013b), specific thresholds separate novel ecosystems from hybrid and historic ones (see box 2). A distinction of novel, hybrid, and historic ecosystem types is particularly useful for defining management goals in the context of restoration ecology. For other research settings, however, it can be helpful to regard ecological novelty as a continuous gradient ranging from historic or analog to novel (see also Hobbs et al. 2013c). Sites can then be ranked with respect to their gradual similarity to a reference state (e.g., Saul et al. 2013, Trueman et al. 2014). Thus, depending on the specific research question, either approach, categorical or continuous, can be more useful.

### Reference conditions

Assessing the degree of novelty of a site or a species’ environment requires a reference. When applying the site-specific perspective, novelty has often been defined by referring to a “historic” state of that site (Hobbs et al. 2006, Hobbs et al. 2013a, Corlett 2015; see Harris et al. 2013 for a discussion of advantages and disadvantages). In the context of biodiversity conservation and ecosystem management, the reference is usually a near-natural system. The late Pleistocene or, in Europe, the last interglacial are often viewed as indicating appropriate reference conditions in discussions about rewilding (Lorimer et al. 2015). However, the human-influenced, pre-industrial conditions sustaining a high biodiversity that were present before the mid-18th century in Europe (e.g., Blackbourne 2006) can also be used as reference. A reference for invasion science frequently is the year 1492 (sometimes rounded to 1500), i.e., when Columbus first arrived in the New World. Particularly in Europe, species that arrived after 1492 are considered neobiota (DAISIE 2009). Reference conditions can also be defined based on recent discussions about the “Anthropocene”. There is increasing evidence that the fundamental, global, and partially irreversible human-induced changes qualify the current era to be classified as a distinct geological epoch (Waters et al. 2016). No consensus about the temporal extent of this period has yet been reached (Ellis et al. 2016), but conditions that existed prior to the onset of this epoch could serve as “historic” reference.

These examples show that reference conditions can be defined in different ways, and misunderstandings can only be avoided by explicitly stating a chosen reference. However, from an evolutionary, palaeontological or biogeographical perspective, change is an inherent feature of life on earth and it seems somewhat arbitrary to define one specific historic state as the baseline. The choice of a reference is influenced by people's background, which varies within and among societies and is also subject to strong temporal changes. People tend to view a particular state of the environment as the “usual” state (e.g., the one they experienced when growing up), and use this state, often unconsciously, as their reference. As a consequence, baselines can shift from one human generation to the following, which in turn can influence conservation goals (Soga and Gaston 2018). Hence, it is vital to provide transparency and justification in choosing reference conditions in novelty approaches. The same is true regarding novelty from an organism-centered perspective. The choice of a reference is part of the first step of every study on ecological novelty (figure 2), and as such is influenced by the social, political, and economic context (see also Backstrom et al. 2018, Prober et al. 2019). Making this choice transparent will enhance communication and subsequent conceptual synthesis of results.

### Intentionality

Most authors frame the definition of concepts on ecological novelty with regard to human activities (Hobbs et al. 2006, Hobbs et al. 2009, Lundholm and Richardson 2010, Hobbs et al. 2013a, Morse et al. 2014, Corlett 2015, Kueffer 2015, Higgs 2017). Yet, there is a debate on which characteristics of human interference are important for the definition. For example, there is no consensus on whether only states resulting from intentional activities should be defined as novel (e.g., draining of wetlands), or also those caused unintentionally (e.g., by global warming or pollution with microplastics). Some authors suggest relating novelty to direct anthropogenic change on a local scale only (Morse et al. 2014, Kattan et al. 2016), whereas others argue that indirect and non-local influences such as pollution can also lead to novelty (Lundholm and Richardson 2010). A related question is whether ecosystems that are managed, for example farmland or managed urban wastelands, should be regarded as novel (e.g., Ellis 2013, Hobbs et al. 2013a, Morse et al. 2014, Kowarik 2017). Our framework of ecological novelty allows for all of these perspectives (figure 1).

Closely related to the question of intentionality is the question of what is meant by “natural”. Naturalness is often used as a reference, or goal, in biodiversity conservation and ecosystem management. The concept of naturalness, however, is quite ambiguous (Siipi 2008), and the same is true for the related concept of wilderness (Kirchhoff and Vicenzotti 2014). For the purpose of building our framework, we limit ourselves to highlighting two different ways in which naturalness can be conceptualized (following Kowarik, 1988, 2017). Classically, naturalness is related to “pristine” conditions, that is, a state preceding major human impact (e.g., Machado 2004). Alternatively, naturalness can be more broadly seen as a state not deliberately designed by people. From this perspective, a natural state can also be reached through a process in which local human intervention has ceased, for example succession on wastelands (Kowarik, 1988, 2017). Novel clearly means the opposite of natural from the first perspective, whereas from the latter perspective, novel ecosystems can also develop towards a natural or “wild” state. Consequently, strongly altered but unmanaged urban areas have sometimes been addressed as “novel wilderness” and have been contrasted to remains of historic ecosystems described as “ancient wilderness” (Kowarik 2017). This conception of naturalness also corresponds to the concept of “wildness” referred to in the context of rewilding (Perino et al. 2019).

**Figure 3.**
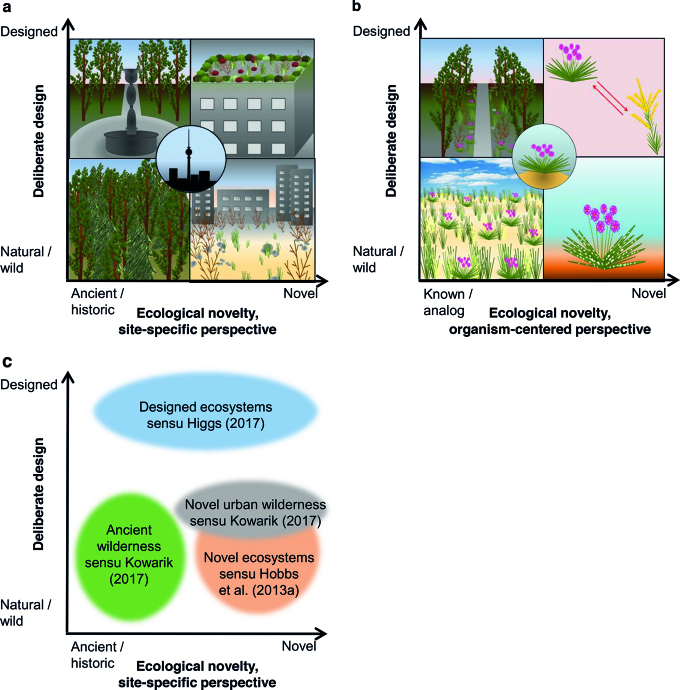
Degree of deliberate design and ecological novelty as two dimensions describing sites and species’ environments in the Anthropocene. Examples are given for (a) the site-specific perspective on ecological novelty with Berlin, Germany, as a focal area (partly based on Kowarik (2017), see Mascaro et al. (2013) for a similar scheme), and (b) the organism-centered perspective, with sea thrift (Armeria maritima) as a focal organism (partly based on Lundholm and Richardson (2010)). Whereas the lower left picture in (b) shows a natural habitat for the focal organism, the upper left picture symbolizes a designed habitat that is analogous to a natural one from the perspective of A. maritima. The upper right picture in (b) symbolizes competition with a neighbor that has been planted, and the lower right picture infestation with a novel pathogen that is unintentionally spreading. (c) Location of some already existing concepts within the conceptual space of deliberate design and ecological novelty.

For the framework of ecological novelty presented here, we adopt the second, broader meaning of natural (that may encompass novelty). We regard a state as natural if it has not been deliberately created by people. A state that has been deliberately created (including maintained) is not natural but designed (Higgs 2017). Conceptually separating deliberate design and ecological novelty allows treatment of these two factors as two dimensions that describe sites or species’ environments in the Anthropocene (figure 3; see also Mascaro et al. 2013, Kueffer and Kaiser-Bunbury 2014). For example, from a site-specific perspective a site can be both designed and ancient (e.g., a historic park), or both natural and novel (e.g., an urban wasteland, figure 3a). Further, from an eco-evolutionary, organism-centered viewpoint, a deliberately designed environment might not be novel. Artificial hard-surfaced urban areas, as one example, resemble rocky habitats; they thus match the eco-evolutionary experience of some cliff plants and mountain birds and can be colonized as an analog habitat (Lundholm and Richardson 2010). Vice versa, ecological novelty can arise due to natural processes. For example, a fungus that spreads without human assistance, that is, naturally, can still cause novelty for a resident organism if it is functionally distinct from the fungi this organism encountered before (figure 3b). Note, however, that the term novel ecosystems has been defined in a much narrower sense (Hobbs et al. 2013a), that is, as ecosystems that develop without human intervention. The term “designed ecosystems” has been suggested to describe ecosystems that are created intentionally and are being maintained and managed to fulfill human needs. Both novel and designed ecosystems can be viewed as novel from a site-specific perspective in our framework (figure 3c).

## Quantifying ecological novelty

Novelty as a cross-disciplinary research domain requires a common “currency”, that is, common measurement units. A straightforward approach for quantifying novelty in many settings is to use established statistical measures for (dis-)similarity or ecological distance (e.g., Bray-Curtis index, standardized Euclidean distance). In the site-specific approach, these indices allow classification of areas as novel or historic, referring to historic species compositions or abiotic conditions (Goring et al. 2016, Leon et al. 2016). They can also be used to quantify a gradient of novelty based on abiotic conditions (Williams et al. 2007) or related proxies (e.g., human population density; Radeloff et al. 2015, Prospere et al. 2016), as well as based on the structure or diversity of communities (Trueman et al. 2014, Prospere et al. 2016).

Dissimilarity indices are also helpful for quantifying novelty from an organism-centered perspective. Saul et al. (2013) proposed a routine for assessing the eco-evolutionary experience (and thus the inverse of novelty) of both introduced and native species with each other. Using a food-web based approach and considering the presence and absence of broadly defined ecological guilds, this routine assesses the ecological similarity between the interaction networks in the native and invaded range. The method can also be applied to plant-pollinator networks, seed-dispersal interactions, and host–parasite systems. An important future direction is the development of corresponding methods to quantify novelty with regard to interactions among further organism groups, for example in plant–plant interactions.

Novelty from an organism-centered perspective can also be measured indirectly by assessing whether the focal organism shows signs of being under increased selective pressure relative to a reference state indicated by a relevant change in morphological, behavioral, or life-history traits. The underlying idea is that a condition that differs from what the species has experienced during its previous evolution will usually exert strong selective pressures (Erfmeier 2013). For all bilaterally symmetric organisms, for example, fluctuating asymmetry (i.e., deviations from perfect bilateral symmetry) is used to monitor environmental stress (Lens and Eggermont 2008). Higher levels of fluctuating asymmetry are considered indicative of stress, and thus can suggest high levels of novelty in the organism's current environment.

Recently, attempts have been made to also assess the ecological effects of different rates and directions of environmental change (Ordonez et al. 2016). Such innovative efforts could allow quantification of the more challenging aspects of ecological novelty in the future, such as interactive effects of different global change drivers and resulting complex and non-equilibrium dynamics.

## Towards management decisions

We introduced ecological novelty as an umbrella concept for the scientific study of ecological and evolutionary effects of human-induced environmental change on organisms, communities, ecosystems, and landscapes. We presented a toolbox for refining the study question (the two perspectives and three qualifiers), and for quantifying ecological novelty. Deriving normative decisions from study results, however, requires leaving the scientific realm, with its ideal of conducting unbiased observations and analyses, and explicitly considering societal values, aims, and processes. Scientific evidence can, for example, tell us whether or not an observation matches the chosen definition of novelty (e.g., a site is novel in comparison to the pre-industrial reference). The evaluation of this result as good or bad, or as tolerable or intolerable, and the potential initiation of management action are beyond the focus of this paper (steps 3 and 4 in figure 2). However, a growing number of decision-support tools are available to facilitate these steps (Hobbs et al. 2014, Backstrom et al. 2018, Prober et al. 2019).

## Future research

We propose the concurrent development of different lines of research on ecological novelty, using two complementary perspectives and three qualifiers, without abandoning established concepts. Instead, our overarching framework integrates these different concepts. In our opinion, diversity in research approaches is needed to account for the complexity of the subject—but leads to the question of how syntheses can be achieved and how different aspects of novelty can be compared across different systems, since synthesizing diverse lines of evidence is a general challenge across research fields (e.g., Lloyd 1994). In this regard, the hierarchy-of-hypotheses (HoH) approach may be a possible way forward (Jeschke and Heger 2018), and developing the concept of ecological novelty into a synthesis approach in which relevant research questions are hierarchically organized and structured should be one aim for the future. With the HoH approach, studies addressing similar questions can be arranged in groups, and conceptual connections of the studies to joint, overarching ideas or hypotheses can be made explicit. Subsequent meta-analyses can then be applied to identify common patterns or causal relationships.

**Glossary**

**Deliberate design:** Intentional alteration of a site to fulfill human benefits. “Designed” is regarded here as the opposite to “natural”, but not to “novel” (see figure 3; see also Lundholm and Richardson 2010, Mascaro et al. 2013, Higgs 2017, Kowarik 2017).

**Ecological novelty:** Umbrella term for addressing consequences of global change for organisms, communities, ecosystems, and landscapes; can be defined from two perspectives:

** Ecological novelty, organism-centered perspective:** A focal species is experiencing a novel environment if the latter differs distinctly from the environment in the focal species’ evolutionary past, i.e., if the focal species lacks eco-evolutionary experience.

** Ecological novelty, site-specific perspective:** The state of a focal area or site is novel if it is distinctly different from a reference specified based on historic criteria (cf. Radeloff et al. 2015), i.e., if the current conditions differ from suggested historic conditions.

**Naturalness:** Aside from other meanings (see e.g., Siipi 2008), two are emphasized here: (a) A state of an area preceding major human impact; here, natural is synonymous with “pristine”; (b) a state of an area that was not deliberately designed and is not maintained by people, that is, it has developed without direct human interference (“naturally”) (Kowarik, 1988, 2017). In this publication, definition (b) is preferred.

**Novel communities:** Combinations of species that have not interacted in their evolutionary past, and that occur because of human-aided shifts in distributions, such as in response to climate change (Tockner et al. 2011, Lurgi et al. 2012).

**Novel ecosystems:** “A novel ecosystem is a system of abiotic, biotic and social components (and their interactions) that, by virtue of human influence, differs from those that prevailed historically, having a tendency to self-organize and manifest novel qualities without intensive human management.” (p. 58 in Hobbs et al. 2013b)

**Novel organisms:** Umbrella term for alien species, range-expanding species, genetically modified organisms (GMOs), synthetic organisms, and emerging pathogens (Jeschke et al. 2013). Novelty is usually considered a consequence of direct or indirect human action, for example the translocation of non-native species. Similar to the term “neobiota” (Kowarik and Starfinger 2009).

**Novel stressor:** Biotic or abiotic component of the environment that has either been created (e.g., synthetic organisms, microplastics, artificial light) or substantially influenced by people (e.g., increased noise level, increased translocation of species leading to biological invasions). This human influence can be intentional or unintentional.

In this paper, we focused on tools for studying effects of human-driven changes in ecological and evolutionary settings at different organizational levels. We did not explicitly consider feedbacks of novelty on society, nor mechanisms by which human activities cause these changes. Figures 1 and 2, however, describe our view on how research on ecological novelty is embedded within social-ecological systems. Future research may aim at a closer integration of the conceptual framework of ecological novelty with the growing field of research on human–nature interactions (cf. Collins et al. 2011, Díaz et al. 2015, Ellis 2015, Perring et al. 2015, Gounand et al. 2018).

## Conclusions

The complexity of current, accelerating environmental changes poses a major challenge for society and science. As a basis for the evaluation of management options, scientific approaches need to cover the consequences of global change for organisms, populations, communities, ecosystems, and landscapes in their entire breadth. The conceptual framework on ecological novelty proposed here (figures 1 and 3, table 1) should advance this urgently needed cross-disciplinary work, for at least three reasons:

1. It allows for a dual focus on sites and on organisms, and thus for the coexistence of complementary ecological and evolutionary perspectives on novelty (figure 1).
2. It facilitates a “common language” (see table 1, figure 1 and the three qualifiers) and joint methods to quantify novelty, and thus will enhance knowledge exchange within and across global-change research (see box 1 for an example) and potentially help resolve controversy about the use of novelty concepts.
3. This common language will also allow for a conceptual integration of research on the consequences of global change for organisms and ecosystems within and beyond biodiversity science, for example by engaging evolutionary ecology, paleoecology, and microbial ecology (box 1). Thus, it will stimulate the integration of research lines on global change that are currently largely separated.

We therefore believe that the multi-faceted framework on ecological novelty proposed here helps in building a broader, integrative basis for a better understanding of the ecological and evolutionary consequences of global change.

## Notes

### Author Biographical

Tina Heger (tina.heger@uni-potsdam.de), Conrad Schittko and Jasmin Joshi are affiliated with University of Potsdam, Biodiversity Research/Systematic Botany, Potsdam, Germany. Tina Heger is also affiliated with the Technical University of Munich, Restoration Ecology, Freising, Germany. Her third affiliation is with the Berlin-Brandenburg Institute of Advanced Biodiversity Research (BBIB), Berlin, Germany; all authors except for Christoph Kueffer share this affiliation. Maud Bernard-Verdier, Monika Hilker, Elisabeth Marquard, Matthias C. Rillig, Wolf-Christian Saul and Jonathan M. Jeschke are affiliated with Freie Universität Berlin, Institute of Biology, Berlin, Germany. Arthur Gessler is affiliated with the Swiss Federal Research Institute WSL, Forest Dynamics, Birmensdorf, Switzerland, and together with Gabriela Onandia also with the Leibniz Centre for Agricultural Landscape Research (ZALF), Müncheberg, Germany. Alex D. Greenwood is affiliated with both the Leibniz Institute for Zoo and Wildlife Research, Berlin, Germany and the Freie Universität Berlin, Department of Veterinary Medicine, Berlin, Germany. Hans-Peter Grossart is affiliated with University of Potsdam, Institute of Biochemistry and Biology, Potsdam, Germany. Together with Wolf-Christian Saul and Jonathan M. Jeschke, he is also affiliated with the Leibniz-Institute of Freshwater Ecology and Inland Fisheries (IGB), Berlin, Germany. Silvia Keinath, Johannes Müller, Stephanie Niemeier and Mark-Oliver Rödel are affiliated with the Museum für Naturkunde – Leibniz Institute for Evolution and Biodiversity Science, Berlin, Germany. Ingo Ko­warik is affiliated with the Technische Universität Berlin, Department of Ecology, Ecosystem Science/Plant Ecology, Berlin, Germany. Christoph Kueffer is affiliated with ETH Zurich, Institute of Integrative Biology, Zurich, Switzerland, and together with Wolf-Christian Saul also with Stellenbosch University, Centre for Invasion Biology (CIB), Department of Botany and Zoology & Department of Mathematical Sciences, Matieland, South Africa. Elisabeth Marquard is affiliated with the UFZ – Helmholtz Centre for Environmental Research GmbH, Department of Conservation Biology, Leipzig, Germany. Jana Petermann is affiliated with the University of Salzburg, Department of Biosciences, Salzburg, Austria. Klement Tockner is affiliated with the Austrian Science Funds – FWF, Vienna, Austria. Jasmin Joshi and Christoph Kueffer are affiliated with the Institute for Landscape and Open Space, HSR Hochschule für Technik, Rapperswil, Switzerland.
